# Modulation of acetate utilization in *Komagataella phaffii* by metabolic engineering of tolerance and metabolism

**DOI:** 10.1186/s13068-019-1404-0

**Published:** 2019-03-21

**Authors:** Qin Xu, Chenxiao Bai, Yiqi Liu, Lili Song, Lin Tian, Yunfeng Yan, Jinfeng Zhou, Xiangshan Zhou, Yuanxing Zhang, Menghao Cai

**Affiliations:** 1 State Key Laboratory of Bioreactor Engineering, East China University of Science and Technology, Shanghai, 200237 China; 2 Shanghai Collaborative Innovation Center for Biomanufacturing, Shanghai, 200237 China

**Keywords:** *Komagataella phaffii*, Acetate utilization, Acetyl-CoA, Kinase screening, Metabolic engineering

## Abstract

**Background:**

Acetate, an economical industrial chemical, which is also the precursor of acetyl-CoA, could serve as an alternative substrate for biomanufacturing. This nontraditional substrate can be widely produced from syngas via hydrolysis or pyrolysis of the cellulosic biomass, chemical or microbial catalysis, anaerobic fermentation in treated wastewater, etc. However, the toxicity of acetate to microorganisms has held back its utilization, especially for the eukaryotes that are good hosts for production of complicated pharmaceuticals or chemicals. This study seeks to improve acetate utilization in a widely used yeast host, *Komagataella phaffii* (previously *Pichia pastoris*), by metabolic engineering of acetate tolerance, transport, and metabolism.

**Results:**

A kinase-deficient library of *K. phaffii* was firstly used to screen acetate-resistant kinases. The *HRK1* knockout strain was sensitive to acetate and overexpression of this gene improved acetate tolerance and cell growth of the strain. Also, overexpression of *HRK1* caused a 55% productivity improvement of acetyl-CoA-dependent 6-methylsalicylic acid (6-MSA). However, activation of Hrk1 on membrane H(+)-ATPase Pma1 seemed not to work in the engineered strain. Acetate transporter gene ScFPS1* was further overexpressed, despite of not improving 6-MSA biosynthesis. To enhance acetate metabolism, acetyl-CoA synthesizing related genes, yeast *PpACS1*, ScACS1*, and *E. coli ackA/pta* were overexpressed separately. Introduction of *PpACS1* and ScACS1* each increased biosynthesis of 6-MSA by approximately 20% on 20 mM acetate. Finally, co-overexpression of *HRK1* and ScACS1* improved 6-MSA productivity by 51% on 20 mM acetate, despite that a low expression level of *HRK1* happened when genes were expressed under the same promoter.

**Conclusions:**

*HRK1* screened by *K. phaffii* kinase-deficient library played an important role in acetate tolerance and was proved to profit the biosynthesis of acetyl-CoA-derived chemicals. It could be a potential target for metabolic engineering of acetate utilization in other eukaryotic hosts as well. A combined strategy of introducing genes for acetate tolerance and metabolism further improved biosynthesis of acetyl-CoA derived reporter compound in *K. phaffii*. This makes it a good choice for acetyl-CoA-derived chemicals with acetate as substrate or precursor in *K. phaffii*, which would also extend the use of this chassis host.

**Electronic supplementary material:**

The online version of this article (10.1186/s13068-019-1404-0) contains supplementary material, which is available to authorized users.

## Background

As promising substrates for industrial biomanufacturing, nontraditional carbon sources, such as acetate, methane, methanol, and syngas, have attracted great attention recently. Acetate (C_2_H_4_O_2_) is one of the simple weak acids, which can be widely produced from syngas via chemical [1] or microbial [2] catalysis or generated from hydrolysis or pyrolysis of the cellulosic biomass [3]. Acetate is also a product of anaerobic fermentation in treated wastewater [4].

Some microorganisms can utilize acetate as a substrate supporting cell growth and metabolism, including the oleaginous yeast *Cryptococcus curvatus* [5, 6], *Escherichia coli* [7, 8], and *Corynebacterium glutamicum* [9]. Acetate can be directly converted into acetyl-CoA, which is catalyzed by cytosolic acetyl-CoA synthetase in eukaryotes such as yeast [10] and by acetate kinase/phosphotransacetylase in prokaryotes like *C*. *glutamicum* [9] and *E*. *coli* [11]. Importantly, acetyl-CoA is a key intermediate of metabolic process in the tricarboxylic acid (TCA) cycle, glyoxylate cycle, and fatty acid synthesis. It also acts as a precursor for many industrially interesting biotechnological products, such as polyketides [12], isoprenoids [13], and lipids [5]. In eukaryotes, acetyl-CoA is compartmentalized in different organelles, which usually limits the conversion efficiency of acetyl-CoA into its derived products [14]. Therefore, acetate may be a promising substrate for enrichment of cytosolic acetyl-CoA and its derived products.

Recently, examples of value-added products derived from acetate by either engineered or natural microbial hosts have been well reported from prokaryotes [7, 8]. An *E. coli* strain with co-overexpression of *acs* (acetyl-CoA synthase gene) and *tes*A (acyl-ACP thioesterase gene) and deletion of *fadE* (acyl-CoA dehydrogenase gene) produced about 1 g/L fatty acids from acetate [8]. The recombinant strain even produced impressive quantities of fatty acids from acetate-rich liquid wastes via dilute acid hydrolysis of lignocellulosic biomass and anaerobic-digested sewage sludge [8]. In another case, succinate production using acetate as the sole carbon source was achieved by modifying the TCA cycle, gluconeogenesis pathway, and glyoxylate shunt in an engineered *E. coli* strain, in which the genes *sdhAB* (encoding succinate dehydrogenase), *iclR* (encoding isocitrate lyase regulator), and *maeB* (encoding malic enzyme) were deleted and *gltA* (encoding citrate synthase) was overexpressed [7]. Some oleaginous yeasts can well utilize acetate, i.e., *C. curvatus* accumulated lipids even up to 73.4% of its dry biomass weight on acetate and glucose [5]. However, acetate easily causes strong antimicrobial effects on the widely used yeast hosts of *Saccharomyces cerevisiae* [15] and *Komagataella phaffii *(previously *Pichia pastoris*) (this study), despite of limited reports regarding metabolic engineering on acetate metabolism in these species [16].

Acetate usually shows antimicrobial action at low pH (< pKa of 4.76) in the undissociated state [15]. In the presence of glucose, undissociated acetate enters cells primarily through the Fps1 aquaglyceroporin channel by facilitated diffusion and dissociates into acetate and proton because of the neutral pH of cytosol in yeast [17]. Acidification of cytoplasm occurs with the accumulation of protons, resulting in the inhibition of important metabolic processes [18] and even programmed cell death [19]. To overcome the stress caused by acetate, Hog1 MAPK is transiently activated in yeast and then phosphorylates Fps1, resulting in Fps1 becoming ubiquitinated, endocytosed, and finally degraded in the vacuole [17]. The degradation of Fps1 is one approach to acetate adaptation in yeast like *S. cerevisiae*. Acetate also leads to activation of the H(+)-ATPase Pma1 located on yeast plasma membrane, which functions to pump protons dissociated by acetate molecules out of cells [20]. Pma1 is crucial for yeast adaptation to acetate, creating an electrochemical proton gradient that is essential for the uptake of nutrients and regulates intracellular pH balance [21, 22]. Moreover, transcription factor Haa1 is essential for rapid adaptation of yeast to acetate, and directly or indirectly regulates approximately 80% of acetate-induced gene expression [23, 24]. Accordingly, the potential for cell death in response to acetate must be considered when developing acetate as substrate.

*Komagataella phaffii* is a versatile and powerful expression host, supporting good expression and bioactivity levels of heterologous proteins [25]. It has been researched for years with mature genetic operation and commercialized vectors and strains [26]. In recent studies, *K. phaffii* has been defined as a good host for biosynthesis of pharmaceutical and chemical molecules derived from acetyl-CoA [27, 28]. Therefore, *K. phaffii* may hold good potential for use in biomanufacturing that converts acetate into a variety of acetyl-CoA-derived [10] and value-added compounds beyond the recombinant proteins.

However, this study finds that *K. phaffii* is quite sensitive to acetate (highly inhibited by acetate over 40 mM, Additional file 1: Fig. S1), which even shows increased sensitivity on comparing with *S. cerevisiae* [17]. We then aim to engineer *K. phaffii* to improve bio-utilization of acetate by metabolic engineering on acetate tolerance, transport and metabolism. As acetate typically causes kinase-related programmed cell death [29, 30], acetate-resistant kinases were screened from a previously constructed *K. phaffii* kinase deletion library [31] and used for construction of acetate-tolerant strains. The reported genes associated with acetate transport [17] and metabolism [32] were overexpressed and their functions in *K. phaffii* were tested. By this means, we dedicate to develop *K. phaffii* strains that can efficiently utilize acetate for production of pharmaceuticals and chemicals.

## Results and discussion

### Screening of acetate tolerance-related genes in *K. phaffii* kinase deletion library

**Fig. 1:**
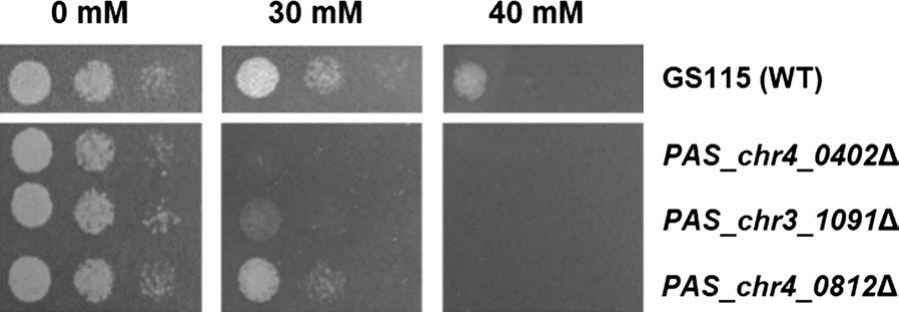
Cell growth of the acetate sensitive knockouts. *K. phaffii* phenotypes of wild-type GS115 (WT), Δ*PAS_chr3_1091* (Δ*HRK1*), Δ*PAS_chr4_0402*, and Δ*PAS_chr4_0812* are shown. Cells were spotted on YPD agar plates (pH 4.5) with acetate of 0, 30 and 40 mM at three concentrations (OD_600_ = 0.1, 0.01 and 0.001). These strains were selected from among the 92 kinase gene knockouts (Additional file 1: Fig. S2)

Toxic levels of acetate may induce programmed cell death (PCD) in yeast, but how the stress signals transmit to cytosol is still not clear yet [29]. Kinases play an important role in cellular signal transduction and are commonly involved in the PCD process [29]. Some kinases, like Hog1, function in acetate transport [29]. There are a total of 152 annotated kinases throughout the genome of *K. phaffii* GS115 [33]. We previously constructed a kinase deletion library consisting of 92 knockout strains of non-essential kinases to screen targets correlated with regulation of methanol metabolism in the methylotrophic yeast *K. phaffii* [31]. Seeing that kinases function in various pathways of cellular signal transduction, we then proceeded to use this kinase deletion library to screen acetate-resistant kinases in this work. Three kinase-knockout strains growing normally without acetate but showing increased sensitivity to acetate (Fig. 1) were screened after examining cell growth on YPD plates supplemented with 0, 30 and 40 mM acetate (Additional file 1: Fig. S2).

Details of annotation and homologs of the screened kinases are summarized in Table 1. The kinase deleted in *PAS_chr3_1091* strain was implicated in activation of the plasma membrane H (+)-ATPase Pma1. Its homolog in *S. cerevisiae* encoded a protein kinase belonging to a family dedicated to the regulation of plasma membrane transporters [30], and it was reported to be possibly involved in the reduction of intracellular acetate concentration [24]. Therefore, *PAS_chr3_1091* is one of the likely targets for acetate tolerance modification. The *PAS_chr4_0402* is annotated as beta regulatory subunit of casein kinase 2, which has a homolog, Ckb1, in *S. cerevisiae* and is related to cell survival [34]. The *PAS_chr4_0812* is annotated as protein serine/threonine/tyrosine kinase, and its homologous gene in *S. cerevisiae* encodes Mck1, playing roles in chromosome segregation and in regulating entry into meiosis [35, 36]. There were also some other knockouts displaying mild sensitivity to high acetate concentrations (Additional file 1: Fig. S2), indicating the complicated response and adaptation of *K. phaffii* cells to acetate stress. As is annotated, the *PAS_chr3_1091* was closely related to acetate-related physiological process [20–22] and was homologous to *HRK*1 in the model yeast of *S. cerevisiae*. Therefore, we identified *PAS_chr3_1091*as *HRK1* and used it as the target for modifying acetate tolerance.

**Table 1:** Kinases related to acetate tolerance screened from *K. phaffii* kinase deletion library [31]

Coding gene	Annotation	Homolog in *S. cerevisiae*
*PAS_chr3_1091*	Protein kinase implicated in activation of the plasma membrane H(+)-ATPase Pma1p	Putative serine/threonine protein kinase Hrk1
*PAS_chr4_0402*	Beta regulatory subunit of casein kinase 2, a Ser/Thr protein kinase	Casein kinase 2 regulatory subunit Ckb1
*PAS_chr4_0812*	Protein serine/threonine/tyrosine (dual-specificity) kinase	Serine/threonine/tyrosine protein kinase Mck1

Besides, deficiency of some kinases caused severe growth defects even in YPD plates without acetate (Additional file 1: Fig. S2). For instance, the *PAS_chr2*-*1_0162* encodes a histidine kinase osmosensor and has a homolog, Sln1, involved in the Hog1 MAP kinase cascade in *S. cerevisiae* [37]. The *PAS_chr2*-*1_0402* is annotated as alpha subunit of heterooctameric phosphofructokinase involved in glycolysis, which has a homolog of Pfk1 in *S. cerevisiae*. The *PAS_chr3_0042* encodes a myristoylated serine/threonine protein kinase involved in vacuolar protein sorting, and its homologous gene encodes Vps15, which is involved in vesicular protein trafficking in *S. cerevisiae* [38]. These kinases are closely related with primary metabolism or global physiological processes, confirming their putative functions as annotated.

### Loss of *HRK1* influences acetate tolerance and cell growth of *K. phaffii*

**Fig. 2:**
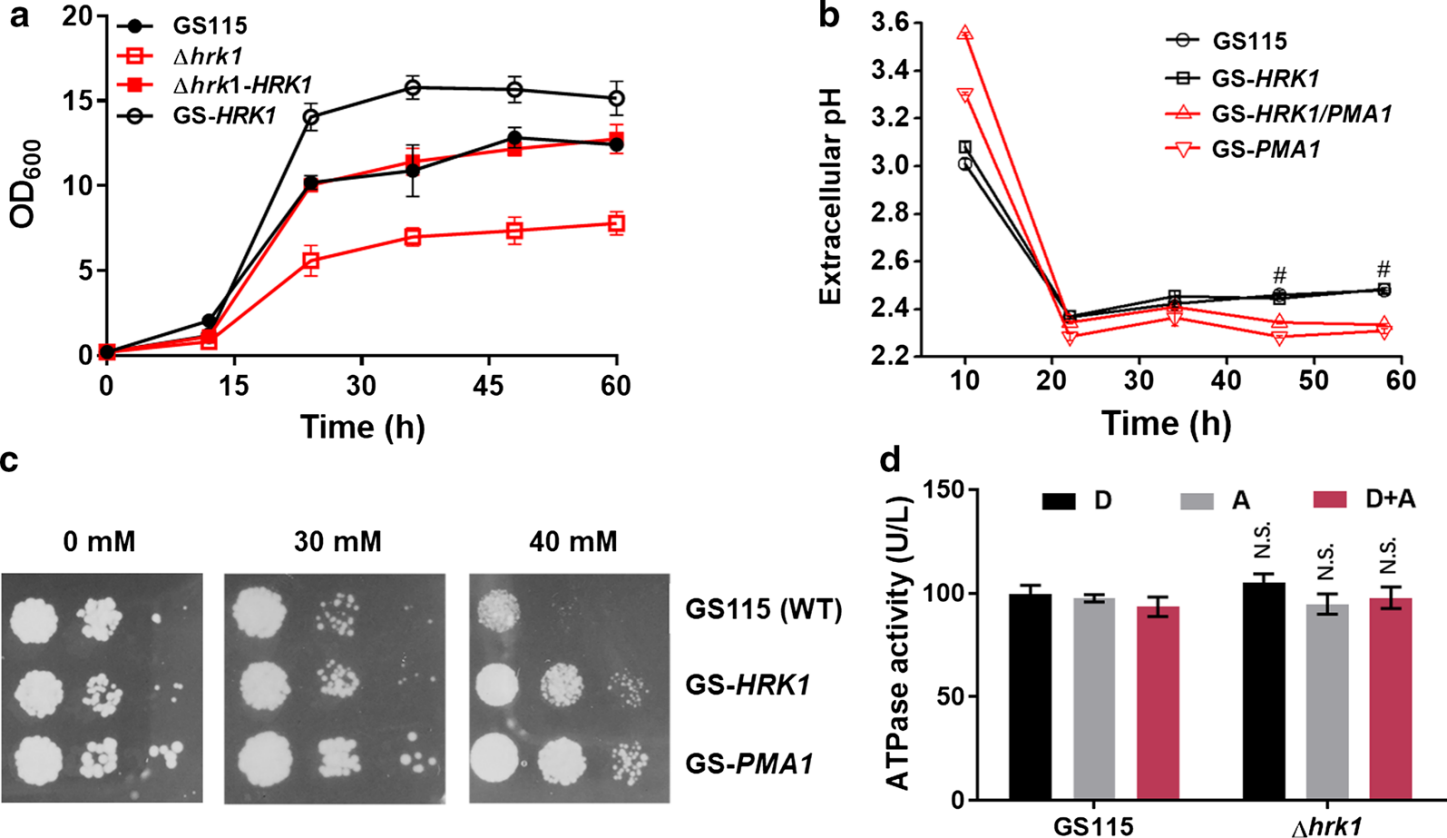
Hrk1 and Pma1 functions in acetate tolerance. **a** Cell growth of *K. phaffii* wild-type GS115 (WT), Δ*hrk1*, Δ*hrk1*-*HRK1* and GS-*HRK1* under YND medium supplemented with 30 mM acetate (medium pH 4.5). **b** The extracellular pH of GS115, GS-*HRK1*, GS-*PMA1* and GS-*HRK1*/*PMA1* in YND medium with initial acetate concentration of 30 mM (medium pH 4.5). ^#^Significant at *P *< 0.05 for GS-*PMA1*&GS115 and GS-*HRK1*/*PMA1*&GS115 at 46 and 58 h, *n *= 6 for each strain. **c** *K. phaffii* phenotypes of wild-type GS115 (WT), *HRK1* overexpression strain (GS-*HRK1*) and *PMA1* overexpression strain (GS-*PMA1*). Cells were spotted on YPD agar plates with acetate of 0, 30 and 40 mM (medium pH 4.5) at three concentrations (OD_600_ = 0.1, 0.01 and 0.001). **d** ATPase activity of GS115 and Δ*hrk* cultivated for 4 h in YND medium (pH 4.5) with different conditions. D, 1% (w/v) glucose; A, 30 mM acetate; D + A, 1% (w/v) glucose and 30 mM acetate. Difference of ATPase activity between Δ*hrk1* and GS115 was not significant (N.S.)

To confirm the role of Hrk1 in acetate tolerance, growth of *K. phaffii* GS115 (wild type) and *HRK1* knockout strain was measured under gradient of acetate concentrations (Additional file 1: Fig. S3). Deletion of *HRK1* caused cell growth of *K. phaffii* to be more sensitive to elevated acetate concentrations (30 and 40 mM). Complementation and overexpression of *HRK1* were then conducted (Additional file 1: Fig. S4). As shown in Fig. 2a, complementation of *HRK1* in Δ*hrk1* largely recovered its growth in the presence of 30 mM acetate. Overexpression of *HRK1* in the wild type further improved cell growth on medium with 30 mM acetate. These results indicated that the kinase encoded by *HRK1* played a critical role in acetate tolerance in *K. phaffii*.

As Hrk1 was implicated in activation of the plasma membrane H(+)-ATPase Pma1 [30], the impact of Hrk1 on Pma1 was then analyzed. As previously reported, Pma1 is a membrane protein in *S. cerevisiae* [39]. Thus, subcellular localization of *K. phaffii* Pma1 was firstly confirmed through a fusion protein of Pma1-green fluorescent protein (GFP). Fluorescence results demonstrated that Pma1 is localized in the membrane in *K. phaffii* (Additional file 1: Fig. S5). Moreover, Student’s *t* test analysis (*P *< 0.05) indicated that extracellular pH of recombinant strains of GS-*PMA1* and GS-*HRK1*/*PMA1* probably differed from that of GS115 and GS-*HRK1* at 46 and 58 h (Fig. 2b). Western blot results showed both *HRK1* and *PMA1* were successfully expressed (Additional file 1: Fig. S4). The results indicated that variation in the extracellular pH was attributable to Pma1 overexpression. In contrast, overexpression of *HRK1* did not affect extracellular pH. However, overexpression of either *HRK1* or *PMA1* enhanced acetate tolerance and cell growth of *K. phaffii* (Fig. 2c). It has been reported that Pma1 is the most abundant protein in the plasma membrane of *S. cerevisiae* and mutation of its phosphorylation sites altered overall ATPase activity in *S. cerevisiae* [40, 41]. Thus, the total ATPase activity was assayed in the WT and Δ*hrk1* cultivated in 1% glucose, 30 mM acetate or their mixture, and the results are presented in Fig. 2d. The ATPase activity of both strains showed no significant difference under the three conditions. Thus, the activation effect of Hrk1 on Pma1 seemed not to work in *K. phaffii*, despite Hrk1 and Pma1 both exhibiting specific functions in regulation of intracellular acetate concentration. However, Hrk1 indeed regulated Pma1 in response to glucose in *S. cerevisiae* [37]. Thus, the results leave open the question of the molecular mechanism of a Hrk1-mediated signal transduction pathway in *K. phaffii*.

### Overexpression of *HRK1* but not *PMA1* promotes biosynthesis of 6-MSA in culture with acetate

As previously reported, a reporter compound derived from acetyl-CoA could be used for evaluation of acetyl-CoA production levels [42]. Acetate is directly catalyzed to acetyl-CoA in yeast; so, quantification of acetyl-CoA-derived products can be used to evaluate the utilization of acetate [42]. Here, we used 6-MSA as a reporter molecule to investigate the effects of various metabolic engineering strategies on acetate utilization in *K. phaffii.* The 6-MSA is a simple and stable polyketide catalyzed from a fungal polyketide synthase [28]. Biosynthesis of 6-MSA uses acetyl-CoA as a starter unit and malonyl-CoA as an extension unit, for which malonyl-CoA is synthesized from acetyl-CoA by acetyl-CoA carboxylase [28]. The 6-MSA biosynthetic pathway, consisting of the *npgA* gene for *Aspergillus nidulans* phosphopantetheinyl transferase and the *atX* gene for *Aspergillus terreus* 6-methylsalicylic acid synthase, was constructed in a *K. phaffii* strain (GS-XN, *abbr.* XN).

**Fig. 3:**
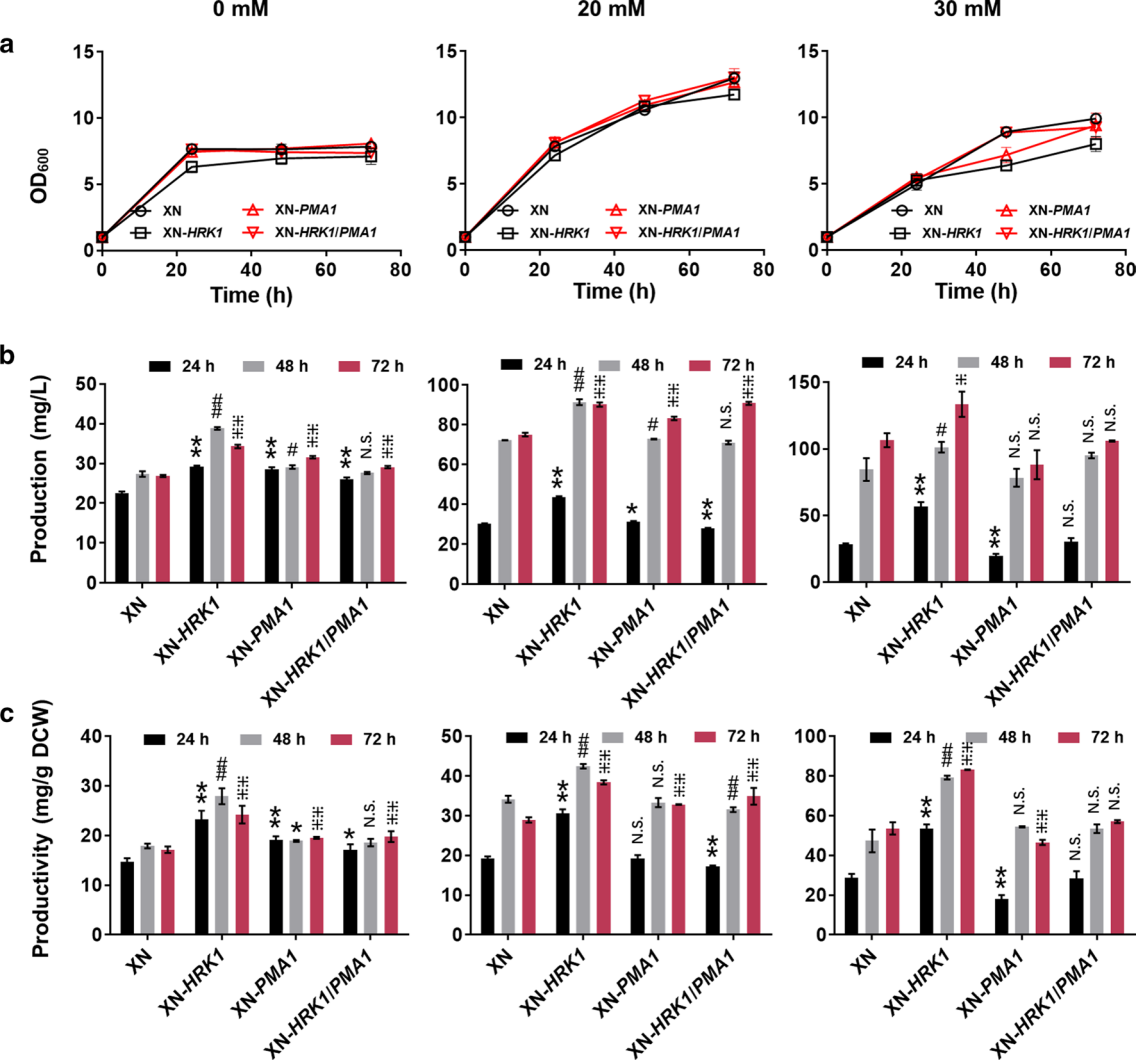
Overexpression of *HRK1* and *PMA1* for 6-MSA biosynthesis. Cell growth (**a**), 6-MSA production (**b**) and 6-MSA productivity (**c**) of recombinant strains XN, XN-*HRK1*, XN-*PMA1* and XN-*HRK1*/*PMA1* in medium with acetate of 0, 20 and 30 mM, respectively. Cells were cultured in YND medium supplemented with 0, 20 or 30 mM acetate (medium pH 4.5) at an initial OD_600_ of 1.0. Then 0.5% (w/v) glucose and acetate with defined levels (20 or 30 mM) were fed every 24 h. Acetate concentrations were marked on top of the figure. Each acetate level was for the group of vertical sub-figures. Statistical significance of 6-MSA production and productivity by overexpression of *HRK1*, *PMA1*, *HRK1*/*PMA1* relative to the parent strain of XN at each time point is also shown. **P < 0.01, *P < 0.05 at 24 h; ^##^*P *< 0.01, ^#^*P *< 0.05 at 48 h; ^⁜⁜^*P *< 0.01, ^⁜^*P *< 0.05 at 72 h; N.S., not significance. *n *= 6 for each strain at specific time points. The error bars represent the standard deviation of three biological replicates assayed in duplicate

Since overexpression of Hrk1 in *K. phaffii* (Fig. 1, Fig. 2) and Pma1 in *K. phaffii* (Fig. 2) and *S. cerevisiae* [43] showed positive effects on acetate tolerance, it was presumed that they might promote acetate metabolism in *K. phaffii*. Thereby, *K. phaffii HRK1* and *PMA1* were overexpressed in the strain XN (Additional file 1: Fig. S6). As shown in Fig. 3a, growth of strains XN, XN-*HRK1*, XN-*PMA1* and XN-*HRK1*/*PMA1* reached stationary phase in 24 h without acetate. However, they could not reach a high cell density as that of the wild-type strain (Fig. 2a) because of the negative effects (antimicrobial) of 6-MSA on cells [44]. Meanwhile, they were still in exponential phase at 24 h in cultures with acetate. Cells in culture with 20 mM acetate reached higher cell density as compared to that without acetate supplementation (Fig. 3a). It indicates that this low level of acetate facilitated cell growth of *K. phaffii* as an effective substrate. Differently, cell growth was not obviously improved by acetate of 30 mM (Fig. 3a), which probably ascribes to the inhibition of cellular metabolic processes by high level of acetate [18]. Expectedly, acetate improved 6-MSA biosynthesis, and particularly, strain XN-*HRK1* showed obvious improvement in production and productivity of 6-MSA under all acetate levels (Fig. 3b, c). The production of 6-MSA in XN-*HRK1* reached the highest level (133.4 mg/L) on 30 mM acetate. Also, productivity of 6-MSA in XN-*HRK1* reached about 83.2 mg/g DCW on 30 mM acetate, 55% higher than that of XN. In contrast, overexpression of *PMA1* had only minor effects on 6-MSA level. Additionally, co-overexpression of *HRK1* and *PMA1* showed positive effects on 6-MSA biosynthesis (Fig. 3b, c) and the protein expression of Hrk1 seems comparable (Additional file 1: Fig. S6). Although overexpression of *HRK1* in wild-type strains promoted cell growth in medium with acetate (Fig. 2a), it did not improve cell growth in the recombinant 6-MSA-producing strain (Fig. 3a).

Generally, overexpression of *PMA1* could not improve 6-MSA biosynthesis in medium with acetate in *K. phaffii,* despite that it resulted in low extracellular pH. Overall, we inferred that Hrk1 plays an important role in acetate tolerance. Pma1, as a plasma membrane H(+)-ATPase that is highly stable and abundant, represents 15% of all plasma membrane proteins in *S. cerevisiae* [42, 43]. Moreover, Pma1 plays multiple roles, such as inducing a constitutive activation of the Hog1 and Slt2 kinases of the high osmolarity glycerol (HOG) and cell wall integrity (CWI) pathways. It is also a major consumer of cellular ATP and has been estimated to consume at least 20% ATP in cells [40, 45]. Therefore, these might affect cell metabolism, which could offset the 6-MSA production improvement attained by overexpression of *HRK1*.

### Introducing acetate transporter ScFps1^*^ is unprofitable to acetate utilization and 6-MSA biosynthesis

A possible limiting step of acetate utilization is the transport of acetate. As previously reported, several plasma membrane transporters are involved in acetate transport, including Ady2 [46], Jen1 [47], and Fps1 [17] in *S. cerevisiae*. Ady2 and Jen1 are responsible for transport of a dissociated form of acetate. However, acetate is substantially undissociated at low pH, which is exactly suitable for the growth of yeast. Fps1 is the membrane channel that facilitates passive diffusional flux of undissociated acetate into the cell in *S. cerevisiae* [17]. The T231A S537A double mutation of *S. cerevisiae* Fps1 (ScFps1^*^) can prevent its in vivo phosphorylation and acetate continuously entering cell [17].

**Fig. 4:**
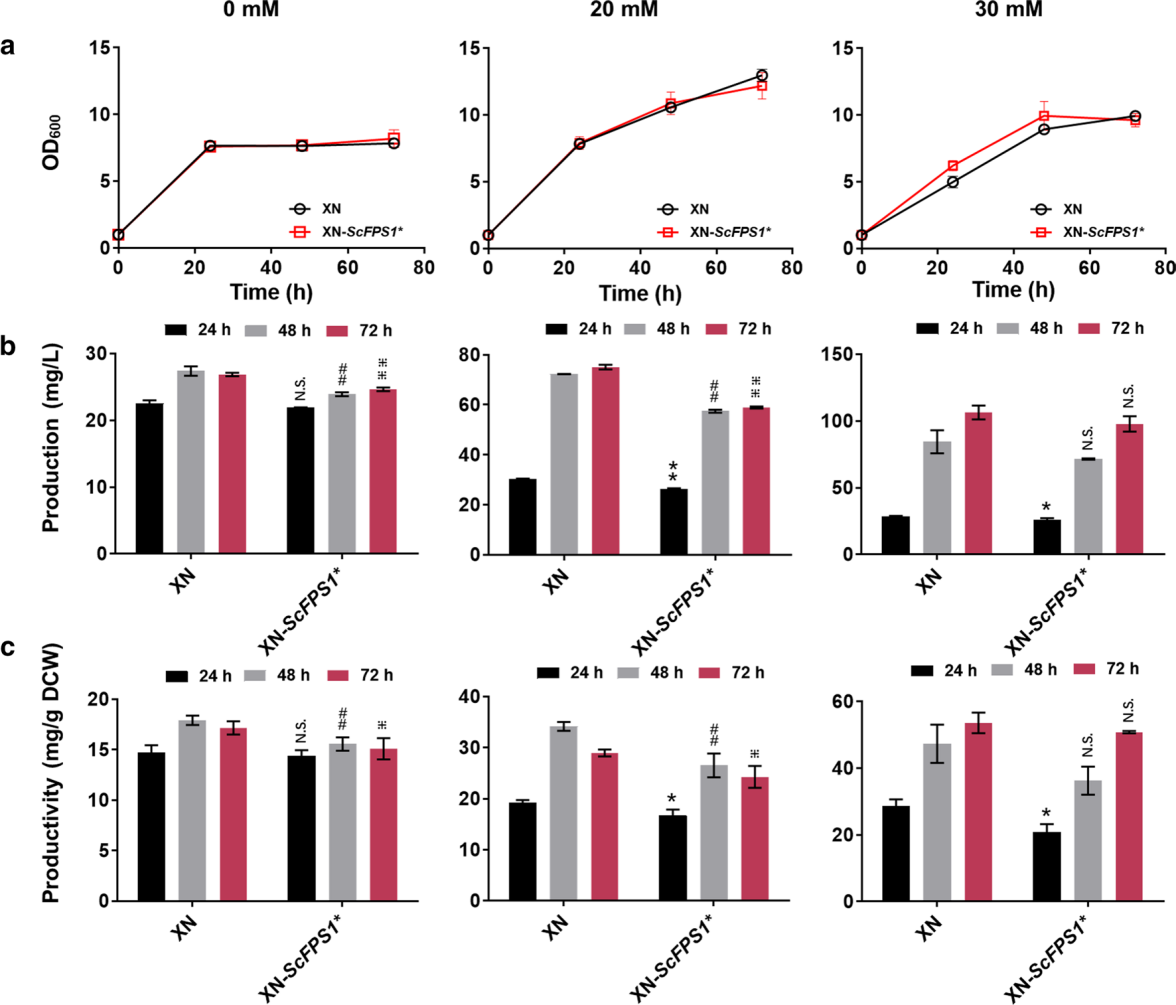
Introducing acetate transport gene ScFPS1* for 6-MSA biosynthesis. Cell growth (**a**), 6-MSA production (**b**) and 6-MSA productivity (**c**) of recombinant strains XN and XN-ScFPS1* in medium with acetate of 0, 20 and 30 mM, respectively. Culture conditions were as same as that described in Fig. 3. Acetate concentrations were marked on top of the figure. Statistical significance of 6-MSA production and productivity by overexpression of ScFPS1* relative to the parent strain of XN at each time point is also shown. **P < 0.01, *P < 0.05 at 24 h; ^##^*P *< 0.01 at 48 h; ^⁜⁜^*P *< 0.01, ^⁜^*P *< 0.05 at 72 h; N.S., not significance. *n *= 6 for each strain at specific time points. The error bars represent the standard deviation of three biological replicates assayed in duplicate

Blast of *S. cerevisiae* Fps1 (GenBank: CAA97494.1) indicates no conserved homolog in *K. phaffii*. Thus, *S. cerevisiae* Fps1 was selected as the target to promote acetate transport and, a recombinant strain (XN-ScFPS1^*^) expressing ScFPS1^*^ (Additional file 1: Fig. S7) was constructed and used for 6-MSA biosynthesis. Cell growth of the XN-ScFPS1* and XN strains were similar in cultures with 0, 20 and 30 mM acetate (Fig. 4a). Overexpression of ScFPS1^*^ did not improve 6-MSA production and productivity, which were even reduced under 0 and 20 mM acetate (Fig. 4b, c). Subcellular localization of ScFps1* and extracellular acetate concentration were further determined. Expression of a fusion protein ScFps1*-GFP indicated that ScFps1* successfully localized within the plasma membrane in *K. phaffii* (Additional file 1: Fig. S5). Nonetheless, broth residual acetate levels of wild-type GS115 and recombinant GS-ScFPS1* remained almost the same throughout the whole process in cultures with an initial acetate concentration of 20 or 30 mM (Additional file 1: Fig. S8). Therefore, we inferred that the native acetate transport ability of *K. phaffii* was sufficient and extra expression of ScFPS1^*^ did not work under these acetate levels. This phenomenon was different from that in *S. cerevisiae* [17]. It was reported that the maximum tolerated concentration of acetate was up to about 100 mM in *S. cerevisiae* (also proved in Additional file 1: Fig. S1) [17, 19], much higher than the 40 mM maximum observed in *K. phaffii* (Additional file 1: Fig. S1). Possibly, the native high acetate transport capacity of *K. phaffii* makes it more sensitive to acetate concentrations, as compared to *S. cerevisiae*.

### Introducing yeast acetyl-CoA synthetases improves acetate utilization and 6-MSA biosynthesis

Since the excessive accumulation of acetate intracellularly can cause damage to cells, it is necessary to engineer a fast and efficient metabolic pathway for acetate in cytosol. It has been reported that acetyl-CoA synthetase (Acs) catalyzes a limiting step due to its low activity and high energy-input requirements [14]. Substituting proline for leucine at position 641 on acetyl-CoA synthetase (SeAcs1) from *Salmonella enterica* could prevent acetylation of SeAcs1 and maintain its high catalytic activity [32]. The amino acid sequences around the acetylation site between *S. enterica* and yeast are well conserved. We then substituted proline for leucine at position 707 on ScAcs1 (GenBank: AAU09675.1) and designated it as ScAcs1^*^. However, there is no conserved acetylation site in PpAcs1 from *K. phaffii*. Afterwards, genes encoding acetyl-CoA synthetase PpAcs1 (NCBI: XP_002491701.1) from *K. phaffii*, ScAcs1^*^ from *S. cerevisiae*, and acetate kinase AckA/phosphotransacetylase Pta with codon-optimized genes from *E. coli* (Additional file 1: Table S1), were separately overexpressed and evaluated in the strain XN (Additional file 1: Fig. S9).

**Fig. 5:**
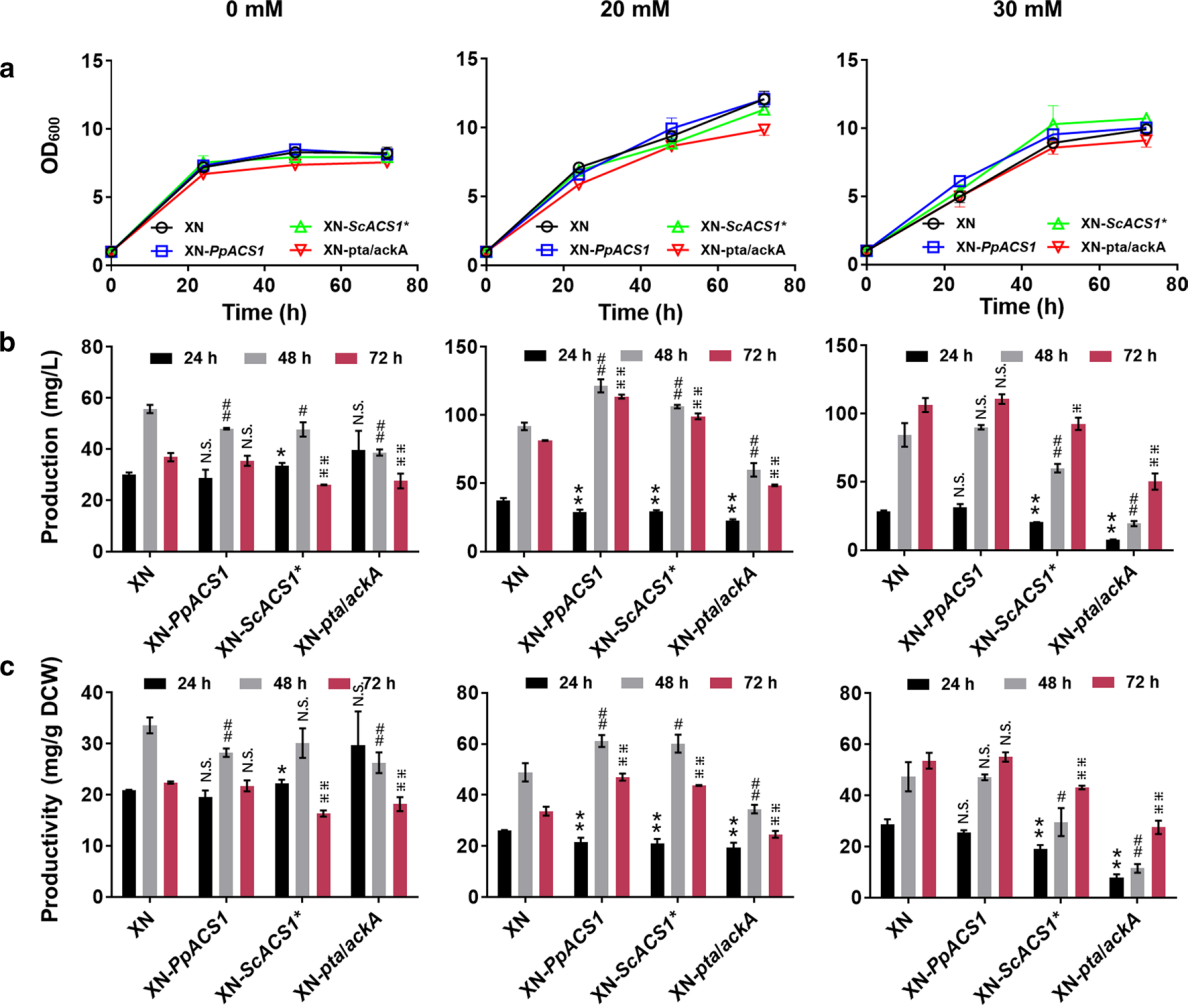
Overexpression of yeast acetyl-CoA synthetase and *E. coli* acetate kinase/phosphotransacetylase gene for 6-MSA biosynthesis. Cell growth (**a**), 6-MSA production (**b**) and 6-MSA productivity (**c**) of recombinant strains XN, XN-*PpACS1*, XN-ScACS1* and XN-*pta*/*ackA* in medium with acetate of 0, 20 and 30 mM, respectively. Culture conditions were as same as that described in Fig. 3. Acetate concentrations were marked on top of the figure. Acetate concentrations were marked the same as those in Fig. 3. Statistical significance of 6-MSA production and productivity by overexpression of *PpACS1*, ScACS1* and *pta*/*ackA* relative to the parent strain of XN at each time point is also shown. **P < 0.01, *P < 0.05 at 24 h; ^##^*P *< 0.01, ^#^*P *< 0.05 at 48 h; ^⁜⁜^*P *< 0.01, ^⁜^*P *< 0.05 at 72 h; N.S., not significance. *n *= 6 for each strain at specific time points. The error bars represent the standard deviation of three biological replicates assayed in duplicate. PpAcs1, acetyl-CoA synthetase from *K. phaffii*. ScAcs1*, acetyl-CoA synthetase from *S. cerevisiae*. AckA, *E. coli* acetate kinase. Pta, *E. coli* phosphotransacetylase

Overexpression of yeast acetyl-CoA synthetase did not affect cell growth (Fig. 5a) but modulated biosynthesis of 6-MSA definitely (Fig. 5b, c). Without acetate feeding, the 6-MSA levels of XN-*PpACS1*, XN-ScACS1^*^ and XN-*Pta*/*AckA* were almost the same as that of XN. However, with 20 mM acetate feeding, biosynthesis of 6-MSA from XN-*PpACS1* and XN-ScACS1^*^ was significantly improved. The 6-MSA productivity of XN-*PpACS1* and XN-ScACS1^*^ achieved about 63.5 and 63.7 mg/g cell at 48 h, respectively, 1.20 and 1.21 times higher than in XN. Nonetheless, overexpression of *PpACS1* or ScACS1^*^ did not improve 6-MSA biosynthesis on 30 mM acetate. Besides, overexpression of *pta*/*ackA* more or less impaired cell growth and biosynthesis of 6-MSA in XN-*pta*/*ackA* also reduced as compared to that in XN under all conditions. As is reported, the acetate kinase/phosphotransacetylase (Ack/Pta) route is reversible. It assimilates acetate in relatively high concentration, as both enzymes possess high *K*_*m*_ values for their substrates in *E. coli* [11]. Thus, acetyl-CoA accumulation might be weakened due to the reversible conversion of acetate into acetyl-CoA, which will further affect cell growth and 6-MSA biosynthesis.

### Co-overexpression of *HRK1* and ScACS1^*^ gives synergy effect

Overexpression of Hrk1, PpAcs1 or ScAcs1^*^ separately has improved utilization of acetate and biosynthesis of its derived polyketide, 6-MSA. Therefore, co-overexpression of *HRK1*/*PpACS1* or *HRK1*/ScACS1^*^ (Additional file 1: Fig. S10) was tested to investigate the possible cooperativity of both enzymes in acetate utilization.

**Fig. 6:**
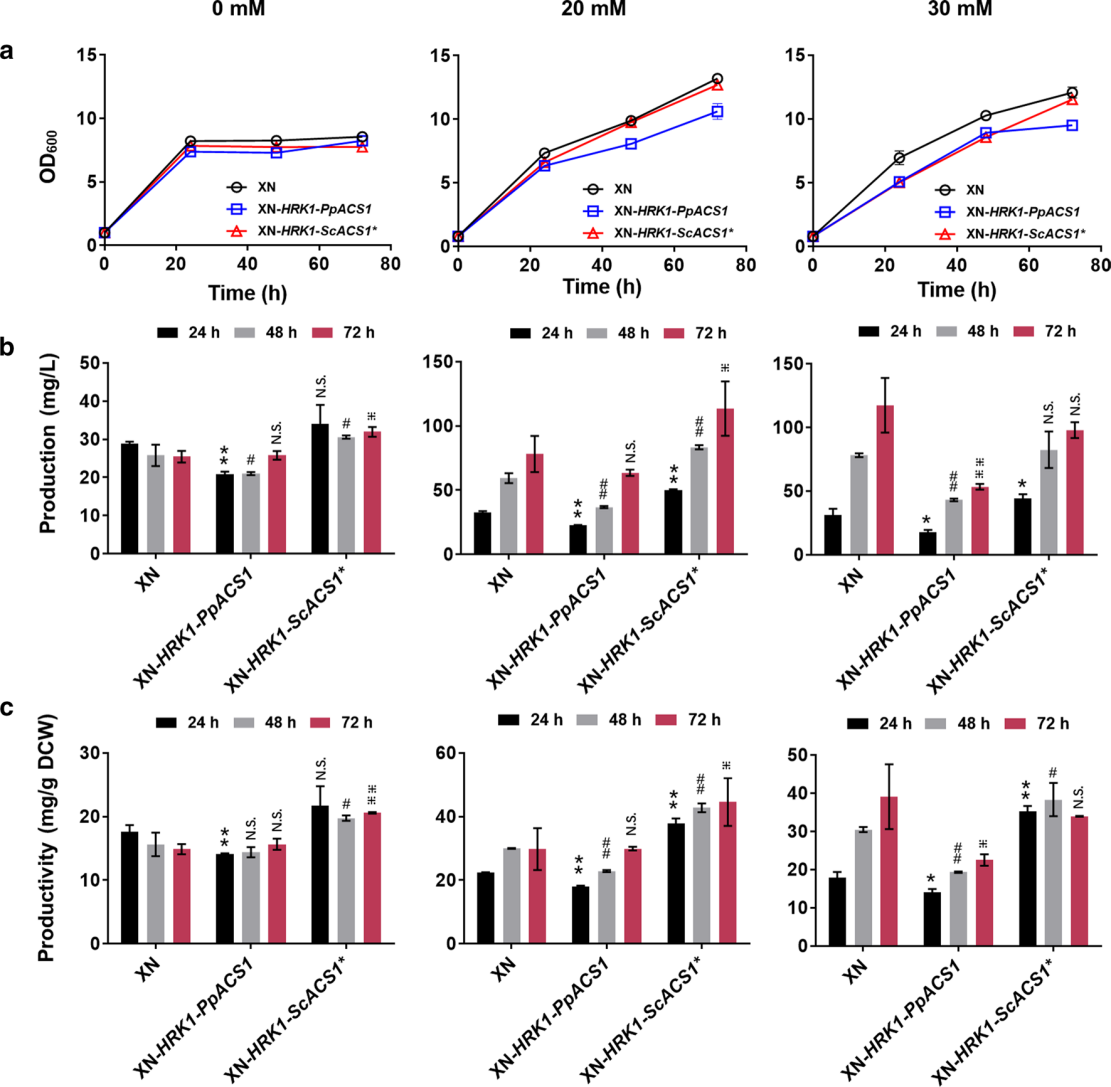
Co-overexpression of *HRK1*/ScACS1*, *HRK1*/*PpACS1* for 6-MSA biosynthesis. Cell growth (**a**), 6-MSA production (**b**) and 6-MSA productivity (**c**) of recombinant strains XN, XN-*HRK1*-*PpACS1* and XN-*HRK1*-ScACS1* in medium with acetate of 0, 20 and 30 mM, respectively. Culture conditions were as same as that described in Fig. 3. Acetate concentrations were marked on top of the figure. Acetate concentrations were marked the same as those in Fig. 3. Statistical significance of 6-MSA production and productivity by overexpression of *PpACS1*, ScACS1* and *pta*/*ackA* relative to the parent strain of XN at each time point is also shown. **P < 0.01, *P < 0.05 at 24 h; ^##^*P *< 0.01, ^#^*P *< 0.05 at 48 h; ^⁜⁜^*P *< 0.01, ^⁜^*P *< 0.05 at 72 h; N.S., not significance. *n *= 6 or 9 for each strain at specific time points. The error bars represent the standard deviation of three biological replicates assayed in duplicate or triplicate

As shown in Fig. 6a, co-overexpression of *HRK1*/*PpACS1* affected cell growth in medium with acetate, as compared to *HRK1*/ScACS1^*^ co-overexpressing strain and the wild type. With 20 mM acetate feeding, biosynthesis of 6-MSA from XN-*HRK1*-ScACS1^*^ highly increased and the production and productivity of 6-MSA reached 113.6 mg/L and 44.6 mg/g DCW (Fig. 6b, c), 45% and 51% higher than the control (XN), respectively. Analyzing the 6-MSA production and productivity ratio of the overexpression strain to its control XN strain in each batch, we may find that co-overexpression of *HRK1*/ScACS1^*^ enhanced 6-MSA biosynthesis, compared with single gene expressing strains of XN-*HRK1* (Fig. 3) and XN-ScACS1^*^ (Fig. 5) under the same acetate feeding concentration of 20 mM. Although individual expression of *PpACS1* and ScACS1^*^ showed similar effects on cell growth and 6-MSA production in medium with acetate, they differed greatly when co-expressed with *HRK1*. An investigation is underway to reveal the background of this phenomenon. Moreover, although co-overexpression of *HRK1*/ScACS1^*^ greatly improved production and productivity of 6-MSA in *K. phaffii* under acetate feeding of 20 mM, the degree of promotion was still lower than that observed with *HRK1* single gene expression strain with 6-MSA production of 133.4 mg/L and productivity of 83.2 mg/g DCW under acetate feeding of 30 mM (Fig. 3b, c). Then, the transcriptional level of *HRK1* in *HRK1*/*PpACS1* or *HRK1*/ScACS1^*^ co-overexpressing strain was analyzed and they both declined compared with the *HRK1* single gene expression strain (Fig. S11). Also, the protein expression of Hrk1 highly decreased in cells of XN-*HRK1*-*PpACS1* and XN-*HRK1*-ScACS1^*^ (Additional file 1: Fig. S10).

As genes expression was driven by *GAP* promoter, increase of heterologous genes controlled by this same promoter will have a ‘titration’ effect on transcription factors, and thus reduce gene expression strength [48, 49]. Thus, the transcription ‘titration’ effect also happened here, which more or less offset the expected synergy improvement effects on 6-MSA biosynthesis. Moreover, as *K. phaffii* cells are sensitive to high acetate concentration, involvement of more heterologous genes and enzymes under this condition will aggravate physiological and metabolic stress, which may further affect 6-MSA biosynthesis. Although Hrk1 plays an important role in acetate tolerance, its function is not implicated in the activation of the plasma membrane H(+)-ATPase Pma1 as its gene annotation describes. Therefore, it is important to search for the possible targets of Hrk1. Clarifying the targets and regulation modes of Hrk1 may provide more ideas for improvement of acetate tolerance in *K. phaffii*. Besides, improvement of acetate metabolism is also crucial to acetate utilization. It not only promotes the metabolism of acetate, but also relieves the adverse effect of acetate to cells to some extent. Our results proved that co-expression of *HRK1* and ScACS1^*^ under the promoter of P_*GAP*_ remarkably improves the acetate utilization. Nonetheless, metabolism of acetate could be further advanced. Use of different promoters to express *HRK1* and ScACS1^*^ and balance their expression to adapt to cells is also necessary to further facilitate acetate utilization in *K. phaffii*.

## Conclusions

*Komagataella phaffii* has shown good potential for use in biomanufacturing, but its high sensitivity to acetate presents problems for the use of acetate as a substrate for acetyl-CoA-derived chemicals. This study finds a native kinase of Hrk1 that plays an important role in acetate tolerance from a kinase-deficient library in *K. phaffii*. It provides a potential target for metabolic engineering of acetate tolerance in eukaryotic expression hosts. Also, improved acetate metabolism can be achieved by overexpression of yeast acetyl-CoA synthetases. Co-overexpression of Hrk1 and acetyl-CoA synthetase ScAcs1^*^ successfully improved production of acetyl-CoA-derived heterologous compound in *K. phaffii*. This work provides reference to the production of pharmaceuticals and chemicals with acetate as carbon source or precursor in *K. phaffii*.

## Methods

### Strains, media and growth conditions

*Escherichia coli* Top10, *K. phaffii* GS115 and expression vectors of pPIC3.5 K and pGAPZαA were purchased from Invitrogen. The pAG32 vector (Hyg^*R*^) was kindly provided by Prof. Saurabh Joshi (University of California, San Diego). *S. cerevisiae* S288c was kindly provided by Prof. Qiang Hua (East China University of Science and Technology).

The *E. coli* strains were grown in light Luria–Bertani (1% [w/v] Bacto peptone, 0.5% [w/v] yeast extract and 1% [w/v] sodium chloride) medium at 37 °C, and 100 μg/mL of ampicillin or 50 μg/mL of Zeocin was added to the medium when required. Yeast for seed preparation was incubated at 30 °C in YPD (2% [w/v] Bacto peptone, 1% [w/v] yeast extract and 2% [w/v] glucose) medium. And 100 μg/mL of Zeocin or 0.75 mg/mL of Hygromycin B was added to the YPD medium when required. 6-Methylsalicylic acid (6-MSA)-producing strains were cultured in synthetic YND medium containing 1.34% (w/v) YNB (yeast nitrogen base without amino acids), 1% (w/v) glucose and acetate with defined levels, and the medium pH was adjusted to 4.5 before sterilization.

### Construction of plasmids and strains

The gene *HRK1* (NCBI: XM_002493292.1), *PMA1* (NCBI: XM_002489588.1) and *PpACS1* (NCBI: XM_002491656.1) were amplified from the genomic DNA of *K. phaffii* GS115. The gene ScFPS1^*^ (NCBI: NC_001144.5) was cloned by three fragments with various primers (GAPZa-Scfps1-F/Scfps1-1(S-A)-R, Scfps1-1(S-A)-F/Scfps1-2(T-A)-R, Scfps1-2(T-A)-F/Scfps1-pGAPZ-R) from *S. cerevisiae* S288c to introduce the mutation of S231A and T537A. The gene ScACS1^*^ (GenBank: AY723758.1) was amplified from *S. cerevisiae* S288c by primers of ScAcs1^*^-pGAP-F/ScAcs1^*^-L-P-pGAPZ-R which contain the mutation of L707P. The gene *ackA* (NCBI: NP_311207.1) and *pta* (NCBI: NP_416800.1) from *E. coli* were codon-optimized and synthesized by Suzhou Genewiz Biotech Co., Ltd., China. The mutant genes of *ScFPS1* and *ScACS1* were denoted as ScFPS1^*^ and ScACS1^*^.

The gene *npgA* (GenBank: AAF12814.1) from *Aspergillus nidulans* and gene *atX* (GenBank: D85860.2) from *Aspergillus terrus* were amplified and cloned into pGAPZαA linearized with *Kpn*Ι/*BspT104*Ι to construct pGAPZ-*npgA* and pGAPZ-*atX*. The plasmid pPICZB-*npgA* was generated by inserting *npgA* expression cassette into pPICZ B linearized with *Bgl*II/*EcoR*Ι. The plasmid pPICZB-*npgA*/*atX* was generated by inserting *atX* expression cassette into pPICZB-*npgA* linearized with *Spe*Ι. The plasmid pPIC-*apgA*/*atX*-*AOX* was generated by inserting *5AOX1* into pPICZB-*npgA*/*atX* linearized with *Spe*Ι. The strain GS-XN (*abbr.* XN) was constructed by transforming linearized plasmid pPIC-*npgA*/*atX*-*AOX* by *pme*Ι into *K. phaffii* GS115 by electroporation.

The plasmids pGAPZ-*HRK1*, pGAPZ-*PMA1*, pGAPZ-*PpACS1* and pGAPZ-ScACS1^*^ were generated by inserting *HRK1*, *PMA1*, *PpACS1* and ScACS1^*^ genes into the vector pGAPZαA linearized with *Kpn*Ι/*BspT104*Ι by a ClonExpress II one step cloning kit (catalog no. C112, Vazyme), respectively. The plasmid pGAPZ-ScFPS1^*^ was generated by assembling three fragments of *ScFPS1* genes with vector pGAPZαA linearized with *Kpn*Ι/*BspT104*Ι by a ClonExpress MultiS one-step cloning kit (catalog no. C113, Vazyme). All these genes assembled with pGAPZαA were under the control of the *GAP* promoter (P_*GAP*_), and the expression cassette of these genes including P_*GAP*_ was amplified by PCR.

The plasmid pAG32-*PMA1* was generated by inserting *PMA1* expression cassette amplified by primers of pAG32DHind3-GAP-F/pAG32BamH1-TT-R into pAG32 linearized with *Hind*III/*BamH*Ι. The plasmid pAG32-*HRK1*/*PMA1* was generated by assembling linearized pAG32-*PMA1* with *Spe*Ι and *HRK1* expression cassette amplified by primers of pAG32-pGAP-F/pAG32-spe1-TT-R. The plasmid pAG32-*5AOX* was generated by inserting 5′ fragment of *AOX1* amplified by primers of pAOX-pAG32Spe1-F/pAOX-pAG32-R with pAG32 linearized with *Spe*Ι to get the integration site for *K. phaffii* genome. The plasmids pAG32-*5AOX*-*HRK1* and pAG32-*5AOX*-ScFPS1* were generated by inserting *HRK1* and ScFPS1^*^ expression cassette amplified by primers of pAG32-pGAP-F/AOXTT-Spe1-pAOX-R with pAG32-5AOX linearized with *Spe*Ι. The plasmids pPIC3.5 K-*PpACS1*, pPIC3.5 K-ScACS1^*^ and pPIC3.5 K-*ackA* were generated by inserting *PpACS1*, ScACS1^*^ and *ackA* expression cassettes amplified by primers of GAP-3.5KSpe1-F/His6-3.5 k-R into pPIC3.5 K linearized with *Sac*Ι*/Not*Ι, respectively. The plasmid pPIC3.5 K-*pta*/*ackA* was generated by inserting *pta* expression cassettes amplified by primers of GAP-3.5 KSpe1-F/GAP-3AOX-R into pPIC3.5 K-*ackA* linearized with *Spe*Ι. The plasmids pAG32-*PMA1*-*gfp* and pAG32-*5AOX*-ScFPS1^*^-*gfp* were generated by inserting *gfp* fragment amplified by primers of pma1-GFP-F/GFP-xhomyc-R and Scfps^*^-GFP-F/GFP-xhomyc-R into pAG32-*PMA1* and pAG32-*5AOX*-ScFPS1* linearized with *Xho*Ι.

The plasmids pAG32-*PMA1* and pAG32-*HRK1*/*PMA1* were linearized by *Stu*Ι, the plasmids pAG32-*5AOX*-*HRK1* and pAG32-*5AOX*-ScFPS1^*^ were linearized by *pme*Ι, the plasmids pPIC3.5K-*PpACS1*, pPIC3.5K-ScACS1^*^ and pPIC3.5K-*pta*/*ackA* were linearized by *BspE*Ι. All these linearized plasmids were transformed into a 6-MSA producing strain GS-XN by electroporation. Meanwhile, linearized pAG32-*HRK1*, pAG32-*PMA1*, pAG32-*HRK1*/*PMA1* and pAG32-ScFPS1^*^ were transformed into *K. phaffii* GS115. Linearized pAG32-*HRK1* was transformed into Δ*hrk1*. The plasmids pAG32-*PMA1*-*gfp* linearized with *Stu*Ι and pAG32-*5AOX*-ScFPS1^*^-*gfp* linearized with *pme*Ι were transformed into *K. phaffii* GS115. All of the plasmids and strains used in this study were summarized in Additional file 1: Tables S2, S3. All of the primers used in this work are summarized in Additional file 1: Table S4.

### Acetate tolerance assay

Strains from the *K. phaffii* kinase deletion library [31] were pre-grown in YPD medium to OD_600_ of 2.0–8.0. Then the cells were harvested by centrifugation at 5000*g* for 3 min and washed three times with sterile water. Acetate solution of 2 M was prepared and pH was adjusted to 4.5 with NaOH. Cell suspensions were spotted 5 μL in three dilutions (OD_600_ of 0.1, 0.01 and 0.001) on YPD agar plates, supplemented with acetate to the final concentration of 0, 30 and 40 mM (medium pH 4.5), respectively. Cell growth was monitored over 2 days at 30 °C.

### ATPase activity assay

Strains of *K. phaffii* GS115 and Δ*hrk1* were pre-grown in YPD medium to OD_600_ of 2.0–8.0; then, the cells were harvested by centrifugation and washed three times with sterile water. Then, cell suspensions were inoculated into YND medium (pH = 4.5) at an initial OD_600_ of 1.0, supplemented with 1% (w/v) glucose, 30 mM acetate or 1% glucose (w/v) + 30 mM acetate, respectively. Cells were harvested after 4-h culture shaking at 200 rpm and 30 °C.

Total protein extracts were purified as described previously [41]. The yeast cells were vortexed with zirconium beads in 25 mM Tris–HCl (pH 8.0), 5 mM EDTA. A protease inhibitor cocktail and a phosphatase inhibitor cocktail (catalog no. P1060, Beyotime) were added prior to cell breakage. The homogenate was cleared from intact cells by centrifugation at 300*g* for 5 min. The supernatant was then centrifuged at 12,000*g* for 30 min. The resulting pellet was suspended in buffer containing 20% (w/v) glycerol, 50 mM Tris–HCl (pH 8), 5 mM EDTA, 2 mM 1, 4-dithioerythritol (DTT) and phosphatase inhibitor cocktail. In those samples used to measure ATPase activity, the phosphatase inhibitor cocktail was omitted in all steps. ATPase activity was measured by ATPase/GTPase activity assay kit (catalog no. MAK113, Sigma-Aldrich) with 2 mM ATP (catalog no. A600020, Sangon).

### Western blot analysis

For each strain, yeast cells (equivalent to cells from 30 mL broth of OD_600_ = 1) were harvested after 32-h culture by centrifugation at 3000*g*, 4 °C for 5 min. The precipitate was washed twice with ice-cold 50 mM binding buffer (10 mmol/L NaH_2_PO_4_, 10 mmol/L Na_2_HPO_4_, 0.5 mol/L NaCl, 20 mmol/L imidazole, pH 7.4), and then resuspended in 1 mL binding buffer. Afterwards, cells were mechanically disrupted by a high-pressure homogenizer for protein analysis. Protein bands were separated by 12% SDS-PAGE and transferred to nitrocellulose membranes. The membranes were incubated with 10% non-fat powdered milk in phosphate-buffered saline containing 0.1% Tween 20 (PBST) for blocking, and then reacted with 6 × His-tag antibody (1:2000 dilution, Beyotime). Secondary antibodies (1:1000 dilution, Beyotime) conjugated with horseradish peroxidase were used and immunoreactive proteins were detected with BeyoECL Plus (Beyotime).

### 6-Methylsalicylic acid analysis

6-Methylsalicylic acid (6-MSA)-producing strains were pre-grown in 50 mL YPD medium to OD_600_ of 2.0–8.0; then the cells were harvested by centrifugation at 3000*g* for 5 min and washed three times with sterile water. The obtained cells were inoculated into YND medium, supplemented with 0, 20 or 30 mM acetate, at an initial OD_600_ of 1.0, and cultured for 72 h. During culture phase, 5 mL broth sample was taken out for analysis every 24 h. Meanwhile, 0.5% (w/v) glucose and acetate with defined levels (20 or 30 mM) were fed into culture broth every 24 h.

After culture for 72 h, 5 ml culture broth was mixed with 10 ml ethyl acetate and vortex oscillated for 2 min. The organic phase was evaporated under reduced pressure and dissolved in methanol. The 6-MSA in extracts was quantified by a high-performance liquid chromatograph (Agilent Technologies 1260 series) equipped with a C_18_ reverse column (Kromasil™, Sweden, 250 mm × 4.6 mm × 5 μm, 100 Å-spherical silica) with a gradient elution strategy at 1 ml/min and detection by UV at 308 nm. Acetate solution (0.1%, phase A) and acetonitrile (100%, phase B) were used as the mobile phase. The sample was subjected to an elution gradient with a mobile phase comprising 25–65% phase A for 20 min followed by 65–100% phase B for 5 min.

### Transcriptional level assays

Strains were pre-cultured in YPD medium, and inoculated into YND medium supplemented with 30 mM acetate, at an initial OD_600_ of 1.0. After culturing for 24 h, yeast cells equivalent to those in 1 mL broth of OD_600_ of 20.0 were harvested by centrifugation. Total RNA of these cells was extracted using yeast total RNA isolation kit (catalog no. B518657, Sangon). Reverse transcription of 1 μg mRNA was performed following FastKing RT Kit (catalog no. KR116, TIANGEN). Real-Time PCR was used to analyze transcriptional level under SuperReal PreMix Plus (SYBR Green) (catalog no. FP205, TIANGEN). The transcriptional level of *HRK1* gene in strain XN-*HRK1* was used as the control.

### Other analytical methods

Cell growth was monitored by measuring OD_600_ using a UV–vis spectrophotometer. Dry cell weight was used for biomass calculation. Cells were centrifugated at 12,000*g* and washed by deionized water for three times. The obtained cells were dried at 70 °C to constant cell weight (dry cell weight). It was measured that OD_600_ of 1.0 makes the equivalent of dry cell weight of 0.2 g/L. Extracellular pH was measured manually by pH meter (catalog no. B-712, Horiba). Acetate level in extracellular supernatant was directly analyzed by enzymatic assays (catalog no. K-ACETRM, Megazyme). Fluorescence of the constructed strains (GS-*gfp*, GS-*PMA1*-*gfp* and GS-ScFPS1*-*gfp*) was observed by inverted microscope DMI3000B (Leica) using a 100× oil immersion objective. Images were processed using Leica application suite (version 2.8.1).

### Statistical analysis

The data were obtained from three biological replicates assayed in duplicate or triplicate, and presented as mean ± S.D. The independent samples student’s *t* test was performed to determine the differences among grouped data. Statistical significance was assessed at *P *< 0.05 and *P *< 0.01.

## Additional file

**Additional file 1.** Additional figures and tables.
