# Supplementary material for: Modulation of acetate utilization in Komagataella phaffii by metabolic engineering of tolerance and metabolism
Source: Biotechnol Biofuels. 2019 Mar 21;12:61. doi: 10.1186/s13068-019-1404-0 (PMC6427870; doi:10.1186/s13068-019-1404-0)
Supplement: Supplementary file 1 — Additional file 1. Additional figures and tables. [file 13068_2019_1404_MOESM1_ESM.docx]

# Additional file 1: Supplementary data

**Modulation of acetate utilization in *Pichia pastoris* by metabolic engineering of tolerance and metabolism**

Qin Xu^1^, Chenxiao Bai^1^, Yiqi Liu^1^, Lili Song^1^, Lin Tian^1^, Yunfeng Yan^1^, Jinfeng Zhou^1^, Xiangshan Zhou^1^, Yuanxing Zhang^1,2^, Menghao Cai^1,*^

^1^ State Key Laboratory of Bioreactor Engineering, East China University of Science and Technology, Shanghai 200237, China

^2^ Shanghai Collaborative Innovation Center for Biomanufacturing, Shanghai 200237, China

*Corresponding author: Menghao Cai

Tel./fax: 86-21-64253306; E-mail: [cmh022199@ecust.edu.cn](mailto:cmh022199@ecust.edu.cn).

**Table S1.** Codon optimized genes for *ackA* and *pta* from *E. coli*

| **Gene** | **Sequence** |
| --- | --- |
| *ackA*  (NCBI: NP_311207.1) | atgtcctctaagttggttttggtccttaactgcggttcctcctccttgaagttcgccatcatcgacgccgtcaacggtgaggagtacttgtctggattggccgagtgtttccatttgccagaggctcgtatcaagtggaagatggacggtaacaagcaggaggctgctttgggagctggtgctgctcactctgaggccttgaatttcattgttaacactatcttggctcagaagccagagttgtccgctcaacttactgccatcggtcacagaatcgtccacggtggtgagaagtacacttcctccgtcgtcatcgatgagtccgtcatccagggtatcaaggacgctgcctctttcgctccattgcataaccctgctcacttgatcggaattgaggaggccttgaagtccttcccacagttgaaggacaagaacgttgctgtttttgataccgcttttcatcagaccatgcctgaggagtcctacctttacgctttgccatacaacttgtacaaggagcacggtatcagaagatacggtgctcacggcacttctcatttctacgttacccaggaggccgccaagatgcttaataagccagttgaggaattgaatattatcacctgccacttgggtaatggaggttccgtttccgctatccgtaacggtaagtgtgttgatacctccatgggtcttaccccattggagggattggttatgggcactagatctggtgacatcgacccagccatcattttccacttgcacgacactttgggtatgtccgttgacgctattaataaattgcttactaaggagtctggtttgcttggtttgaccgaggtcacttccgattgcagatacgtcgaggacaactacgctaccaaggaggacgccaagagagctatggacgtctactgccatcgtttggccaagtacatcggtgcttacaccgccttgatggacggtagattggacgccgttgttttcaccggtggaattggtgagaacgctgctatggtcagagagttgtccttgggaaagttgggtgtcttgggattcgaggttgatcacgaacgtaacttggccgccagatttggtaagtccggtttcatcaacaaggagggcactagaccagccgttgtcattcctaccaacgaagagttggtcatcgctcaagacgcctctagattgactgct |
| *pta* gene (NCBI: NP_416800.1) | atgtcccgtattattatgctgatccctaccggaaccagcgtcggtctgaccagcgtcagccttggcgtgatccgtgcaatggaacgcaaaggcgttcgtctgagcgttttcaaacctatcgctcagccgcgtaccggtggcgatgcgcccgatcagactacgactatcgtgcgtgcgaactcttccaccacgacggccgctgaaccgctgaaaatgagctacgttgaaggtctgctttccagcaatcagaaagatgtgctgatggaagagatcgtcgcaaactaccacgctaacaccaaagacgctgaagtcgttctggttgaaggtctggtcccgacacgtaagcaccagtttgcccagtctctgaactacgaaatcgctaaaacgctgaatgcggaaatcgtcttcgttatgtctcagggcactgacaccccggaacagctgaaagagcgtatcgaactgacccgcaacagcttcggcggtgccaaaaacaccaacatcaccggcgttatcgttaacaaactgaacgcaccggttgatgaacagggtcgtactcgcccggatctgtccgagattttcgacgactcttccaaagctaaagtaaacaatgttgatccggcgaagctgcaagaatccagcccgctgccggttctcggcgctgtgccgtggagctttgacctgatcgcgactcgtgcgatcgatatggctcgccacctgaatgcgaccatcatcaacgaaggcgacatcaatactcgccgcgttaaatccgtcactttctgcgcacgcagcattccgcacatgctggagcacttccgtgccggttctctgctggtgacttccgcagaccgtcctgacgtgctggtggccgcttgcctggcagccatgaacggcgtagaaatcggtgccctgctgctgactggcggttacgaaatggacgcgcgcatttctaaactgtgcgaacgtgctttcgctaccggcctgccggtatttatggtgaacaccaacacctggcagacctctctgagcctgcagagcttcaacctggaagttccggttgacgatcacgaacgtatcgagaaagttcaggaatacgttgctaactacatcaacgctgactggatcgaatctctgactgccacttctgagcgcagccgtcgtctgtctccgcctgcgttccgttatcagctgactgaacttgcgcgcaaagcgggcaaacgtatcgtactgccggaaggtgacgaaccgcgtaccgttaaagcagccgctatctgtgctgaacgtggtatcgcaacttgcgtactgctgggtaatccggcagagatcaaccgtgttgcagcgtctcagggtgtagaactgggtgcagggattgaaatcgttgatccagaagtggttcgcgaaagctatgttggtcgtctggtcgaactgcgtaagaacaaaggcatgaccgaaaccgttgcccgcgaacagctggaagacaacgtggtgctcggtacgctgatgctggaacaggatgaagttgatggtctggtttccggtgctgttcacactaccgcaaacaccatccgtccgccgctgcagctgatcaaaactgcaccgggcagctccctggtatcttccgtgttcttcatgctgctgccggaacaggtttacgtttacggtgactgtgcgatcaacccggatccgaccgctgaacagctggcagaaatcgcgattcagtccgctgattccgctgcggccttcggtatcgaaccgcgcgttgctatgctctcctactccaccggtacttctggtgcaggtagcgacgtagaaaaagttcgcgaagcaactcgtctggcgcaggaaaaacgtcctgacctgatgatcgacggtccgctgcagtacgacgctgcggtaatggctgacgttgcgaaatccaaagcgccgaactctccggttgcaggtcgcgctaccgtgttcatcttcccggatctgaacaccggtaacaccacctacaaagcggtacagcgttctgccgacctgatctccatcgggccgatgctgcagggtatgcgcaagccggttaacgacctgtcccgtggcgcactggttgacgatatcgtctacaccatcgcgctgactgcgattcagtctgcacagcagcag |

**Table S2.** List of plasmids used in this study.

| **Plasmids** | **Characteristic(s)** | **Reference** |
| --- | --- | --- |
| pPIC3.5 K | Ampicillin^R^; G418^R^; HIS4; P_AOX1_-based yeast expression vector | Invitrogen |
| pGAPZαA | Zeocin^R^; P_GAP_-based expression yeast vector | Invitrogen |
| pAG32 | Ampicillin*^R^*; hygromycin*^R^* | Ref. [1] |
| pGAPZ-*PMA1* | pGAPZαA derivative containing *P. pastoris* *PMA1* gene | This study |
| pGAPZ-*HRK1* | pGAPZαA derivative containing *P. pastoris HRK1* gene | This study |
| pGAPZ-*ScFPS1^*^* | pGAPZαA derivative containing *S.cerevisiae ScFPS1^*^* gene | This study |
| pGAPZ-*PpACS1* | pGAPZαA derivative containing *P. pastoris* *PpACS1* gene | This study |
| pGAPZ-*ScACS1^*^* | pGAPZαA derivative containing *S.cerevisiae* *ScACS1^*^* gene | This study |
| pGAPZ-*ackA* | pGAPZαA derivative containing *ackA* gene | This study |
| pGAPZ-*pta* | pGAPZαA derivative containing *pta* gene | This study |
| pPIC3.5 K-*PpACS1* | pPIC3.5 K derivative containing P*_GAP_*-*PpACS1* expression cassette | This study |
| pPIC 3.5 K-*ScACS1^*^* | pPIC3.5 K derivative containing P*_GAP_*-*ScACS1^*^* expression cassette | This study |
| pPIC 3.5 K-*ackA* | pPIC3.5 K derivative containing P*_GAP_*-*ackA* expression cassette | This study |
| pPIC 3.5 K-*pta*/*ackA* | pPIC3.5 K derivative containing P*_GAP_*-*ackA*  and P*_GAP_*-*pta* expression cassette | This study |
| pAG32-*PMA1* | pAG32 derivative containing P*_GAP_*-P*MA1* expression cassette | This study |
| pAG32-*5AOX-HRK1* | pAG32 derivative containing P*_GAP_*- *HRK1* expression cassette | This study |
| pAG32-*5AOX-ScFPS1^*^* | pAG32 derivative containing P*_GAP_*- *ScFPS1^*^* expression cassette | This study |
| pAG32-*HRK1*/*PMA1* | pAG32 derivative containing P*_GAP_*-*PMA1* and P*_GAP_*-*HRK1* expression cassette | This study |
| pAG32-*PMA1-gfp* | pAG32 derivative containing P*_GAP_*- *PMA1-gfp* expression cassette | This study |
| pAG32-*5AOX-ScFPS1^*^-gfp* | pAG32 derivative containing P*_GAP_*- *ScFPS1^*^-gfp* expression cassette | This study |

**Table S3.** List of strains used in this study.

| **Strains** | **Genotype** | **Reference** |
| --- | --- | --- |
| *Escherichia coli* Top 10 | F^-^[*lacI*^q^ Tn*10*(Tet^r^)] *mcrA* Φ80*lacZ* ΔM15 Δ*lac X74* *deoR* *recA1* | Invitrogen |
| *Pichia pastoris* GS115 | *his4* | Invitrogen |
| GS-XN | GS115 with plasmid pPIC ZB-*atX-npgA* | This study |
| GS-*HRK1* | GS115 with plasmid pAG32- *HRK1* | This study |
| Δ*hrk1* | GS115 *PAS_chr3_1091*Δ::*Sh ble his4* | Ref. [2] |
| Δ*hrk1*-*HRK1* | Δ*hrk1* with plasmid pAG32-*HRK1* | This study |
| GS-*PMA1* | GS115 with plasmid pAG32-*PMA1* | This study |
| GS-*HRK1*/*PMA1* | GS115 with plasmid pAG32-*HRK1*/*PMA1* | This study |
| GS-*ScFPS1*^*^ | GS115 with plasmid pAG32-*ScFPS1^*^* | This study |
| XN-*HRK1* | GS-XN with plasmid pAG32-*HRK1* | This study |
| XN-*PMA1* | GS-XN with plasmid pAG32-*PMA1* | This study |
| XN-*HRK1*/*PMA1* | GS-XN with plasmid pAG32-*HRK1*/*PMA1* | This study |
| XN-*ScFPS1*^*^ | GS-XN with plasmid pAG32-*ScFPS1^*^* | This study |
| XN-*PpACS1* | GS-XN with plasmid pPIC3.5 K-*PpACS1* | This study |
| XN-*ScACS1*^*^ | GS-XN with plasmid pPIC3.5 K-*ScACS1*^*^ | This study |
| XN-*pta*/*ackA* | GS-XN with plasmid pPIC3.5 K-*pta*/*ackA* | This study |
| XN-*HRK1-PpACS1* | XN-*HRK1* with plasmid pPIC3.5 K-*PpACS1* | This study |
| XN-*HRK1*-*ScACS1*^*^ | XN-*HRK1* with plasmid pPIC3.5 K-*ScACS1*^*^ | This study |
| GS-*PMA1-gfp* | GS115 with plasmid pAG32-*PMA1-gfp* | This study |
| GS-*ScFPS1*^*^*-gfp* | GS115 with plasmid pAG32-*5AOX*- *ScFPS1*^*^*-gfp* | This study |

**Table S4** List of primers used in this study^a^.

| **Primers** | **Sequence (5’ to 3’)** |
| --- | --- |
| hrk1-pGAPZ-F | TCAATCAATTGAACAACTATATGCCATCTAAGTTTTTATCATTC |
| hrk1-pGAPZ-R | CTGGCGGCCGCCGCGGCTCGAGGTGCAGATGGCACTGC |
| pma1-pGAPZ-F | TCAATCAATTGAACAACTATATGTCCGCTGAAGAGCC |
| pma1-pGAPZ-R | CTGGCGGCCGCCGCGGCTCGAGGACCAGACTTCTCGTGCTG |
| GAPZa-Scfps1-F | TCCCTATTTCAATCAATTGAACAACTATATGAGTAATCCTCAAAAAGCTC |
| Scfps1-1(T-A)-R | CAAGACTGTAGGAGCTTGAGGGTTCTGGTATAATG |
| Scfps1-1(T-A)-F | CAGAACCCTCAAGCTCCTACAGTCTTGCCCTCC |
| Scfps1-2(S-A)-R | CCAGTTGACTGGAGCTTCATGACCCTGATAAATACAG |
| Scfps1-2(S-A)-F | CAGGGTCATGAAGCTCCAGTCAACTGGTCTTTAC |
| Scfps1-pGAPZ-R- | CTGGCGGCCGCCGCGGCTCGAGGTGTTACCTTCTTAGCATTACCATAAT |
| PpAcs1-pGAP-F | TTTCAATCAATTGAACAACTATATGCCATTAGATAACGAACACTT |
| PpAcs1-pGAPZ-R | AGCTGGCGGCCGCCGCGGCTCGAGGTTTGCGGGCATCCCTTTTAAC |
| ScAcs1*-pGAP-F | TCAATCAATTGAACAACTATATGTCGCCCTCTGCCG |
| ScAcs1*-L:P-pGAPZ-R | GCGGCCGCCGCGGCTCGAGGCAACTTGACCGAATCAATTGGATGTC |
| pAG32-pGAP-F | TCATCGATGATATCAGATCAGATCTTTTTTGTAGAAATGTCTTGG |
| AOXTT-Spe1-pAOX-R | ACCTTTCGTCTTTGGATGTTGACTAGTCTCTCACTTAATCTTCTGTACTCTG |
| pAG32DHind3-GAP-F | GAACGCGGCCGCCAGCTGAGATCTTTTTTGTAGAAATGTCTTGG |
| pAG32BamH1-TT-R | GCGCGCCTTAATTAACCCGGGGATCCTCTCACTTAATCTTCTGTACTCTGAA |
| pAG32-spe1-TT-R | GATCCGCGGCCGCATAGGCCACTAGTTCTCACTTAATCTTCTGTACTCT |
| pAOX-pAG32Spe1-F | TCATCGATGATATCAGATCCACTAGTAACATCCAAAGACGAAAGGTT |
| pAOX-pAG32-R | GATCCGCGGCCGCATAGGCCCGTTTCGAATAATTAGTTGTTTTTTGA |
| GAP-3.5KSpe1-F | CCAGTTATTGGGCTTGATTGGACTAGTCAGATCTTTTTTGTAGAAATGTCT |
| His6-3.5k-R | AACAGTCATGTCTAAGGCGAATTAATTCTCAATGATGATGATGATGATGGTC |
| GAP-3AOX-R | CAAGACATTTCTACAAAAAAGATCTTCTCACTTAATCTTCTGTACTCTGA |
| Scfps1-GFP-F | GTAATGCTAAGAAGGTAACAACCATGGGTTCTAAAGGTG |
| GFP-xhomyc-R | AAAGCTGGCGGCCGCCGCCTATTTGTACAATTCATCCATACC |
| pma1-GFP-F | CTCAGCACGAGAAGTCTGGTACCATGGGTTCTAAAGGTG |

^a^Restriction enzyme digestion sites are underlined.


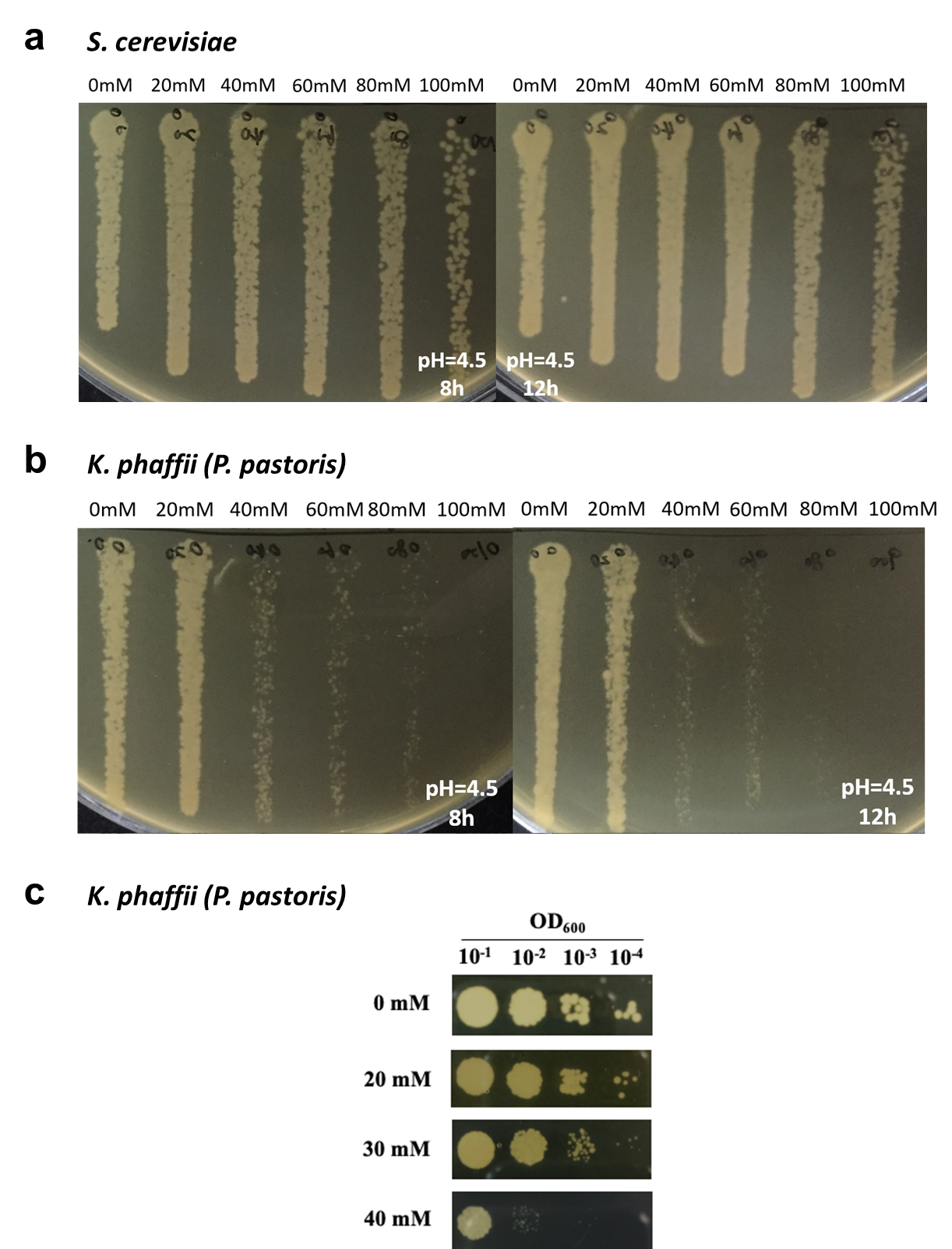


**Fig. S1** Cell growth of wild type yeasts on YPD plates with various acetate levels. (a) *K. phaffii* (*P. pastoris*) GS115 cells were firstly prepared following the acetate tolerance assay in the Methods. Then cells were inoculated into YPD medium supplemented with various levels of acetate (medium pH 4.5) to OD_600_ of 1.0. After cultured for 8 h and 12 h, cells were diluted to OD_600_ of 1.0 by YPD medium supplemented with the corresponding levels of acetate (medium pH 4.5). Then 15 µl diluted cells were spotted and flowed through YPD plate, and keep it in the super-clean bench until broth dried. Then the plates were cultured at 30℃ for 2 days. (b) *Saccharomyces cerevisiae* S288c. Culture methods referred to *K. phaffii*. (c) Cell suspensions were spotted 5 μl in dilutions (OD_600_ of 0.1, 0.01, 0.001 and 0.0001) on YPD agar plates supplemented with acetate to the final concentration of 0, 20, 30 and 40 mM (medium pH 4.5), respectively. Cells were cultured at 30℃ for 2 days.


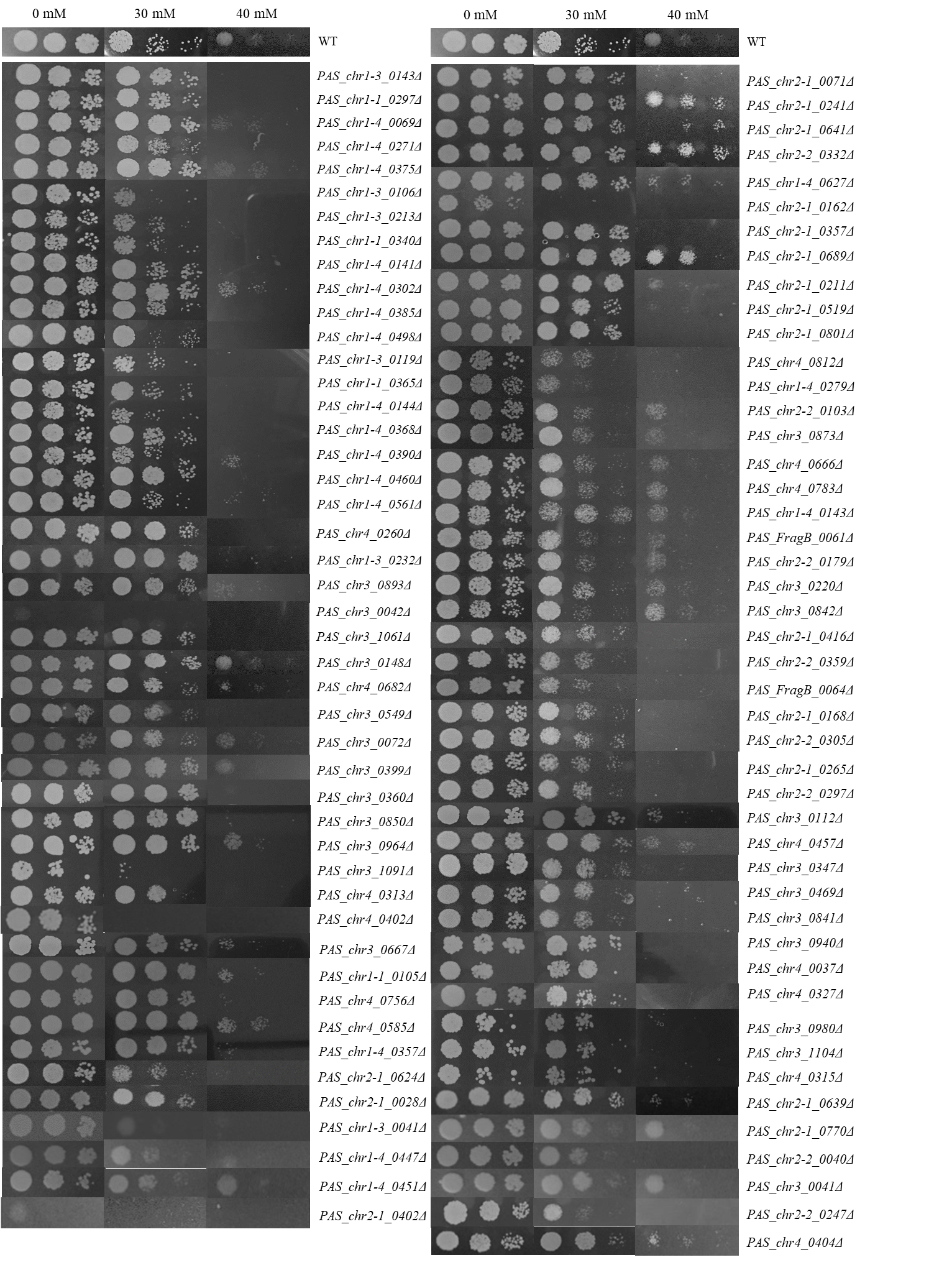


**Fig. S2** Cell growth of 92 knockouts of kinase genes on acetate. Cells were cultured on YPD plates (pH 4.5) for 2 days with 0, 30 and 40 mM acetate, respectively. Three cell densities (OD_600_=0.1, 0.01 and 0.001) were used for spottings. Strains were constructed in our previous study [2].


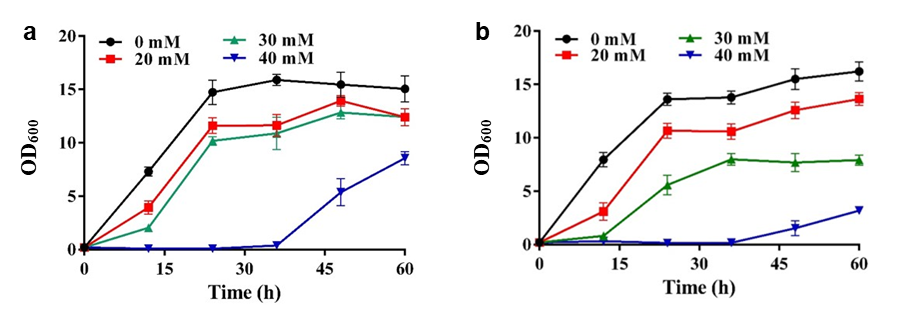


**Fig. S3** Cell growth of *P. pastoris* wild type (GS115) and Δ*hrk1*. (a) GS115; (b) Δ*hrk1* mutant. Cells were grown in YND medium (pH 4.5) with initial 0, 20, 30 and 40 mM acetate.


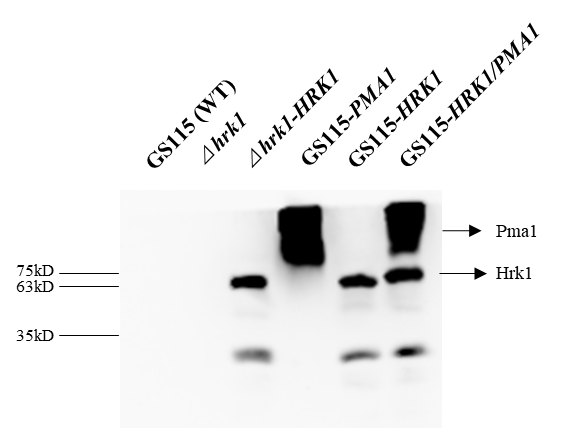


**Fig. S4** Western blotting analysis of different strains described in Fig. 2**.** GS115 (WT), wild type *P. pastoris* GS115; Δ*hrk1*, *HRK1* deficient strain; Δ*hrk1*-*HRK1*, complementation of *HRK1* in Δ*hrk1*; GS115-*PMA1,* overexpression of *PRM1* in *P. pastoris* GS115; GS115-*HRK1,* overexpression of *HRK1* in *P. pastoris* GS115; GS115-*HRK1/PMA1,* overexpression of *HRK1* and *PMA1* in *P. pastoris* GS115. Theoretical molecular weight: Pma1, 97.89 kD; Hrk1, 66.21 kD.


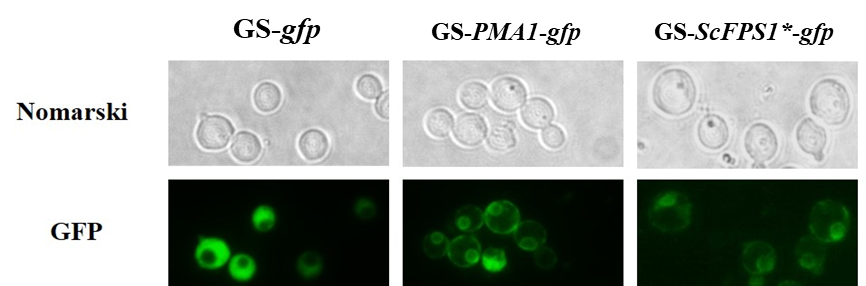


**Fig. S5** Subcellular localization of plasma membrane protein Pma1 and Scfps1*. Strains of GS-*gfp*, GS-*PMA1*-*gfp* and GS-*ScFPS1**-*gfp* were cultivated to logarithmic phase, and then inoculated to YPD medium with initial OD_600_ of 1.0 for 4 h at 30℃. Yeast cells were washed with deionized water before observing.


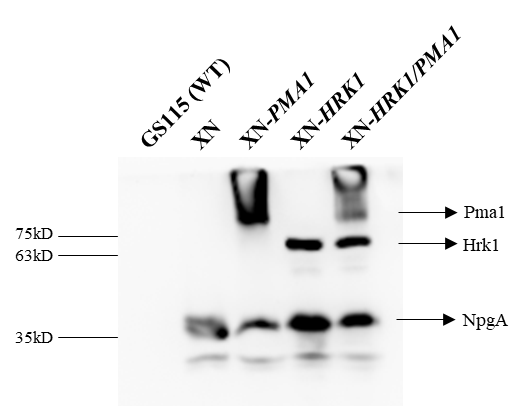


**Fig. S6** Western blotting analysis of different strains described in Fig. 3**.** GS115 (WT), wild type *P. pastoris* GS115; XN, recombinant *P. pastoris* GS115 producing 6-methylsalicylic acid; XN-*PMA1*, overexpression of *PRM1* in XN; XN-*PMA1*, overexpression of *HRK1* in XN; XN-*PMA1*, overexpression of *HRK1/PRM1* in XN. Theoretical molecular weight: Pma1, 97.89 kD; Hrk1, 66.21 kD; NpgA, 37.73 kD. The ‘smearing’ effect of Pma1 may ascribe to the cell debris of the membrane protein of Pma1.


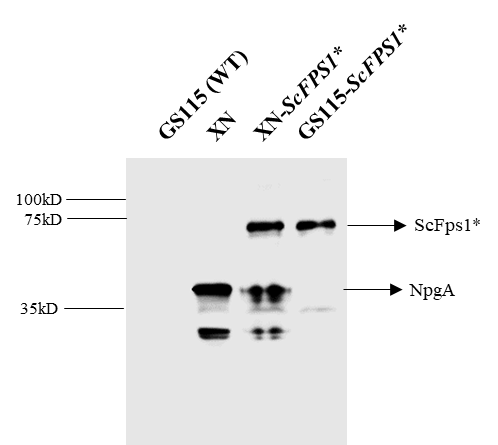


**Fig. S7** Western blotting analysis of different strains described in Fig. 4. GS115 (WT), wild type *P. pastoris* GS115; XN, recombinant *P. pastoris* GS115 producing 6-methylsalicylic acid; XN-*ScFPS1^*^*, overexpression of *ScFPS1^*^* in XN; *GS115*-*ScFPS1^*^*, overexpression of *ScFPS1** in *P. pastoris* GS115. Theoretical molecular weight: ScFps1^*^, 73.89 kD; NpgA, 37.73 kD.


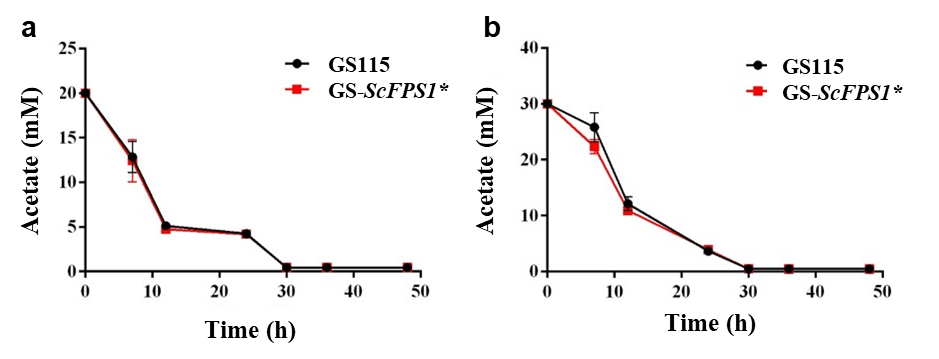


**Fig. S8** Extracellular acetate concentration of wild type (GS115) and GS-*ScFPS1** strains on 20 mM (**a**) and 30 mM (**b**) acetate. Unsignificant at *P*>0.05 for GS115&GS-*ScFPS1** at 7 h and other time points under 30 mM acetate.


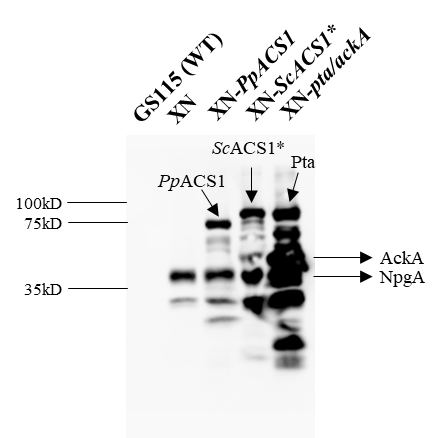


**Fig. S9** Western blotting analysis of different strains described in Fig. 5. Western blotting analysis of different strains described in Fig. 3**.** GS115 (WT), wild type *P. pastoris* GS115; XN, recombinant *P. pastoris* GS115 producing 6-methylsalicylic acid; XN-*PpACS1*, overexpression of *P. pastoris ACS1* in XN; XN-*ScACS1^*^*, overexpression of *S. cerevisiae ACS1^*^* in XN; XN-*pta/ackA*, overexpression of *pta/ackA* in XN. Theoretical molecular weight: Pta, 77.18 kD; ackA, 43.3 kD; PpAcs1, 73.93 kD; ScAcs1^*^, 79.15 kD; NpgA, 37.73 kD.


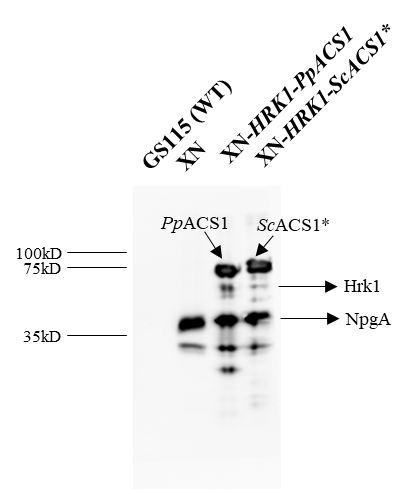


**Fig. S10** Western blotting analysis of different strains described in Fig. 6. Western blotting analysis of different strains described in Fig. 3**.** GS115 (WT), wild type *P. pastoris* GS115; XN, recombinant *P. pastoris* GS115 producing 6-methylsalicylic acid; XN-*HRK1*-*PpACS1*, overexpression of *P. pastoris ACS1* and *HRK1* in XN; XN-*HRK1*-*ScACS1^*^*, overexpression of *S. cerevisiae ACS1^*^* and *HRK1* in XN. Theoretical molecular weight: PpAcs1, 73.93 kD; ScAcs1^*^, 79.15 kD; NpgA, 37.73 kD; Hrk1, 66.21 kD.

**Fig. S11** Transcriptional levels of *HRK1* in different strains cultured in YND medium (initial pH 4.5) with 30 mM acetate for 24 h. Relative mRNA levels of *HRK1* in XN-*HRK1* on 30 mM acetate was used as the control. The relative expression level indicated on the *y* axis (2^-△△Ct^) for each gene was normalized to *HRK1* in XN-*HRK1*.

**Reference**

1. Waterham HR, Digan ME, Koutz PJ, Lair SV, Cregg JM. Isolation of the *Pichia pastoris* glyceraldehyde-3-phosphate dehydrogenase gene and regulation and use of its promoter. Gene. 1997;186:37-44.

2. Shen W, Kong C, Xue Y, Liu Y, Cai M, Zhang Y, et al. Kinase screening in *Pichia pastoris* identified promising targets involved in cell growth and alcohol oxidase 1 promoter (P*_AOX1_*) regulation. PloS One 2016;11:e0167766.
